# Activin is a neural inducer of a male-specific muscle in *Drosophila*

**DOI:** 10.1038/s41598-024-54295-3

**Published:** 2024-02-14

**Authors:** Ken-ichi Kimura, Rimi Kumano, Daisuke Yamamoto

**Affiliations:** 1 Laboratory of Biology, Sapporo Campus, Hokkaido University of Education, Sapporo, 002-8502 Japan; 2 Advanced ICT Research Institute, National Institute of Information and Communications Technology, Kobe, 651-2492 Japan

**Keywords:** Sexual dimorphism, Development

## Abstract

*Drosophila melanogaster* has a pair of male-specific muscles called the muscle of Lawrence (MOL) in abdominal segment 5 (A5) of adult flies. The MOL is produced only when its innervating motoneuron expresses FruitlessM (FruM) neural masculinizing proteins. We show that MOL induction is hampered by: (1) silencing electrical activities in the motoneuron, (2) blocking vesicular release from the motoneuron, and (3) knocking down Activin ß (Actß) in the motoneuron or knocking down Actß signaling pathway components in the myoblasts. Our timelapse live imaging of the developing neuromuscular system reveals that, upon contact with the presumptive MOL, the motoneuronal axon retracts concomitant with the progression of MOL degeneration resulting from neural silencing. We conclude that MOL formation depends on the bidirectional trophic interactions between pre- and postsynaptic cells, with motoneuron-derived Actß playing an inducing role in MOL formation.

## Introduction

Sex-dependent divergence in behavior is commonly seen across the animal kingdom. Such behavioral sex differences primarily stem from sexually dimorphic neural circuit structures and functions^1–3^. In rodents, sex differences in mating behavior and aggression have been traced back to sexually dimorphic projections and connectivity patterns within molecularly defined neuronal populations in the hypothalamus and associated subcortical nuclei^4–6^. In humans, sexually dimorphic anatomy in the hypothalamus has also been implicated in the gendered expression of psychophysiological and behavioral traits^7–10^. Neural sexual dimorphism extends beyond the hypothalamus and is prevalent throughout the entire nervous system of vertebrates. This includes the peripheral nervous system, which offers a convenient avenue for experimental intervention to investigate its mechanistic underpinnings. For example, neurons in the spinal nucleus of the bulbocavernosus (SNB), a sexually dimorphic (male-enlarged) motor center involved in penile erection in males, innervate the levator ani and bulbocavernosus muscles, both of which are important for copulatory actions: a perinatal androgen surge occurring specifically in males stimulates the muscles to produce a neurotropic factor, which is then incorporated into SNB motoneurons, preventing their degeneration^11,12^. Circulating androgens later in life elaborate dendrites of SNB motoneurons. Within the mammalian SNB system, the precise dynamics of interactions between neurons and muscles have not been well defined, and the identity of the neurotropic factor involved remains obscure.

In the genetic model organism *Drosophila melanogaster*, neural sexual differences have been extensively explored with single-cell resolution^13^. Sex steroids such as androgens and estrogens are detected only at trace levels in insects, including Diptera^14^. Instead, transcription factor proteins produced by two major sex-determination genes, *fruitless* (*fru*) and *doublesex* (*dsx*), are the key players in the sexual differentiation of neurons in *Drosophila*^15^. Whereas *dsx* is involved in the sex determination across a wide variety of cell types of both somatic and germline lineages^16,17^, *fru* plays a masculinizing role specifically in a subset of neurons^18,19^. This specificity of *fru*’s actions enables one to manipulate a limited number of well-defined neural cells without interfering with the functions of the majority of cells in the body. The *fru* gene has at least four promoters: the distalmost P1 promoter produces transcripts encoding male-specific proteins FruM (Fru male-specific), which are responsible for the sex-determinant actions of the *fru* gene^19,20^. Other promoters serve general developmental roles in both sexes^21,22^. FruM proteins include five isoforms‒AM, BM, CM, DM, and EM, each of which has a different C-terminus. Paradoxically, the functions of these isoforms are distinct, in that each has unique binding targets, and also overlapping, as some isoforms phenotypically compensate for the lack of other isoforms^23,24^. FruM proteins are transcription factors and thus potentially affect a wide range of biological processes, depending on the transcriptional targets they act on. Two firmly established targets of FruM are *roundabout1* (*robo1*) and *teiresias* (*tei*), both belonging to IgG superfamily members known to function as cell recognition molecules. In fact, *robo1* and *tei* mediate the sex-specific shaping of neurite structures of *fru*-expressing neurons^25,26^. FruM proteins also control sex-specific cell death, likely regulating the transcription of some cell-death genes, resulting in the sex-dependent presence or absence of certain neurons and the sex-dependent abundance of particular neuron types^27,28^. In addition to these cell-autonomous actions, FruM proteins are known to exert a cell non-autonomous action: FruM expression in a presynaptic motoneuron is required for the normal development of a postsynaptic muscle cell, a male-specific muscle called the muscle of Lawrence (MOL; 20). The MOL is a dorsal longitudinal muscle present in abdominal segment 5 (A5) of the adult male fly^29,30^. Although its function is obscure, the MOL offers an ideal platform for investigating the mechanistic basis for inductive interactions between cells for the formation and maintenance of the synapse. This is because both presynaptic and postsynaptic cells can be identified as single cells, and the peripheral localization of the neuromuscular synapse offers easy access for experimental intervention. Previous studies demonstrated that motor innervation triggers the aggregation of myocytes, ultimately leading to the development of myotubes and the formation of the MOL^31^. This occurs only when the motoneuron is genetically male (X/O or X/Y), irrespective of whether the recruited myocytes are female (X/X) or male^32,33^. However, the nature of presynaptic inductive signals (e.g., electrical activities per se or specific molecules) has remained unclarified, and possible retrograde influences of the postsynaptic muscle on the presynaptic motoneuron have not been explored. In this study we attempt to address these questions by interfering with the action potential conduction via overexpression of the inward rectifier K^+^ channel *Kir2.1*^34^, chemical transmission via *shi*^*ts*^ mutant transgene expression^35^ or putative inducer signaling via RNAi-based knockdown of genes^36^ for representative signaling molecules and their transduction mediators. We show that, for the MOL to form, the innervating motoneuron must be electrically active, vesicular release from presynaptic terminals needs to be functional, and activin must be derived from the motoneuron to act on myoblasts that contribute to the MOL. Our timelapse live imaging of the developing neuromuscular system revealed that, once the motoneuronal axon makes contact with the presumptive MOL, it retracts in tandem with the progression of MOL degeneration resulting from neural silencing. This contrasts with motoneurons innervating conventional muscles, which persist even when their electrical activities are silenced. These observations strongly suggest that dynamic bidirectional interactions^37^ play crucial roles in the formation and maintenance of the male-specific muscle MOL.

## Results

**Figure 1:**
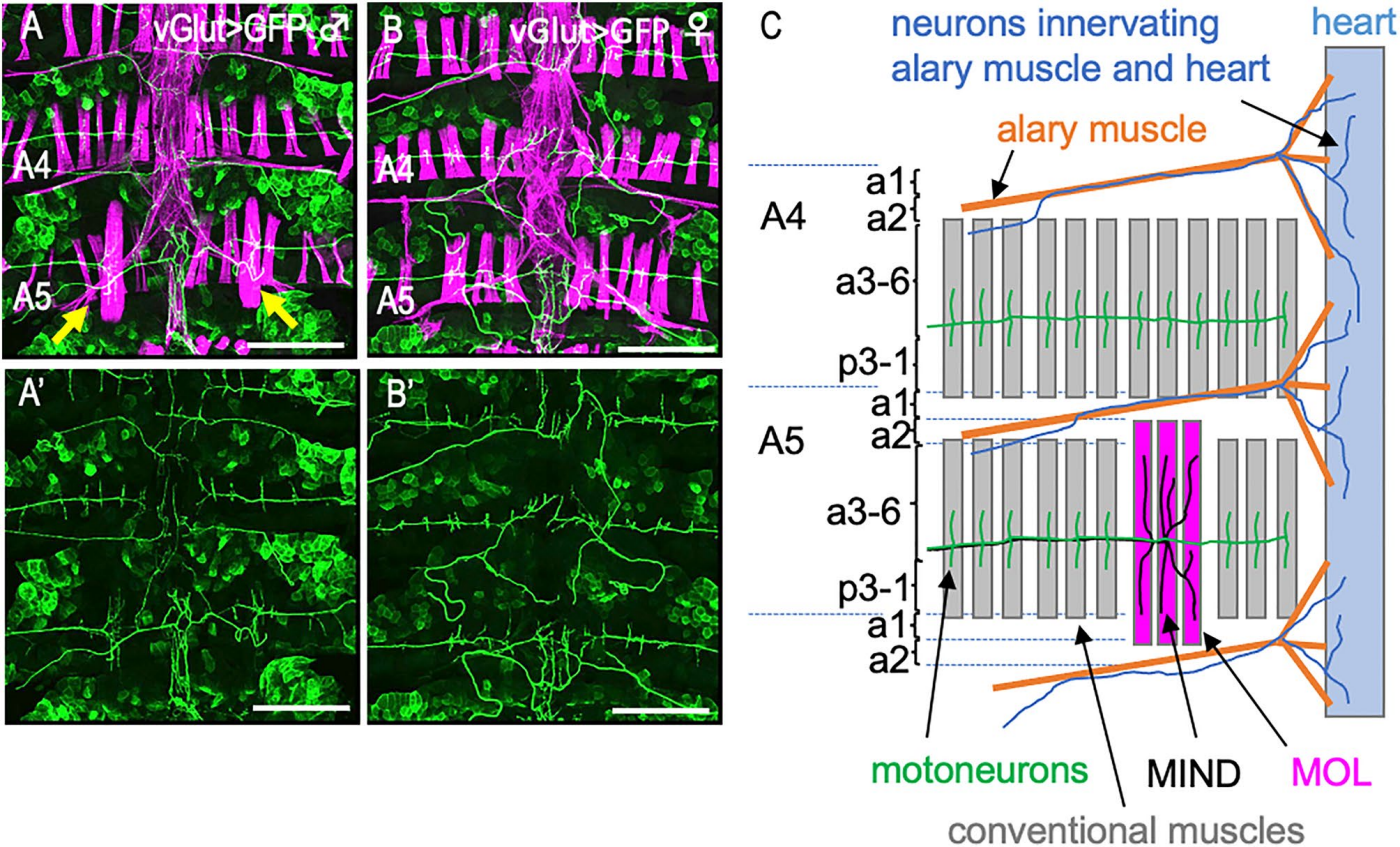
The male-specific muscle MOL and its innervation by the Mind neuron. (**A**–**B’**) Dorsal musculatures (**A** and **B**; magenta) and innervating nerves (**A**-**B’**; green) of adult male (**A**, **A’**) or female (**B**, **B’**) abdominal segments 4 (A4) and 5 (A5). (**C**) A schematic drawing of the nerve and muscle layout is shown for the A4 and A5 hemi-segments. Cuticular subdomains (a1 to p3-1) are also indicated. The MOLs are indicated by yellow arrows in (**A**). The MOLs varied in appearance around their attachment sites from individual to individual, particularly when observed with high contrast imaging: some appeared to be split into a few bundles. The MOLs are indicated by yellow arrows. Muscles were stained with phalloidin and nerves were labeled with GFP. Note that the phalloidin-labeled fibers which run across the segments are heart muscles, the phalloidin-labeled elements that irradiate diagonal thin fibers are of alary muscles, and fat bodies are occasionally labeled with GFP. The genotype of the flies is *y hs-flp; vGlut*^*OK371*^*-GAL4 UAS-mCD8::GFP*. Scale bar: 200 µm.

The fully developed MOL is readily distinguishable from other muscles by its large size (Fig. 1A,B). More specifically, the MOL’s attachment sites to the cuticle are shifted in the anteroposterior axis; the anterior end of the MOL attaches to the middle of a2 in the A5 segment and the posterior end to a2 in the A6 segment. This is unlike conventional muscles, whose anterior attachment site is near the border between a2 and a3 and whose posterior attachment site is localized in a1 of the next segment A6 (Fig. 1C; Ref.^38^). Here a2 and a3 are the two of nine cuticular subdomains in each adult segment defined by the surface structure, pigmentation, bristles and Hh-responsiveness^38,39^. The MOL is also distinct in that it highly expresses 79B-Actin, which is also present in conventional longitudinal muscles, though at trace levels^40,41^. MOL formation depends on FruM expression in the innervating motoneuron. Consequently, males that carry a null mutation in the *fru* locus lack the MOL^29^. The MOL-innervating motoneuron that expresses FruM has been identified by a single-cell clonal analysis and named the MOL-inducing motoneuron (abbreviated as the Mind motoneuron; Ref.^42^]. When FruM is overexpressed under the control of *D42-GAL4*, a pan-motoneuronal driver, additional MOLs are produced in other abdominal segments in addition to the normal MOL in A5 in males and, in the case of females, ectopic MOLs are produced in A5 and often in other abdominal segments, too^20^.

**Figure 2:**
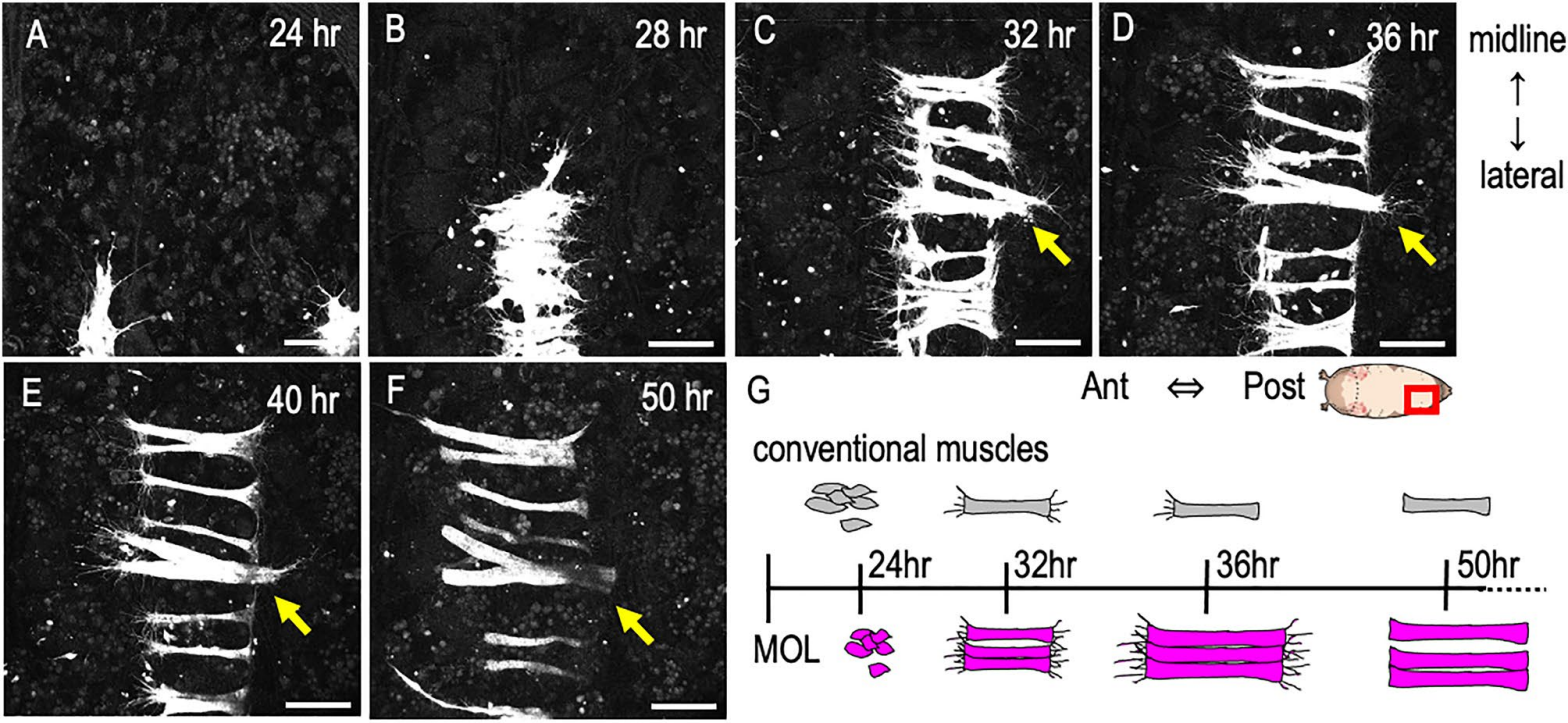
Dynamics of the developing MOL. (**A**–**F**) Snapshots of myogenesis in A5 captured every 4 h between 24 and 50 h apf. The midline is on the upper side, while the lateral edge is on the lower side. Proliferated myoblasts move toward the midline and fuse with each other to form an elongated myotube (**A**,**B**). The myotube actively protrudes filopodia-like structures while extending toward an apodeme (**C**). Conventional muscles extend to the apodeme at their posterior end, where they cease protruding filopodia-like structures (**D**). Subsequently, the anterior end extends to the apodeme (**E**). The MOL continues to grow, attaining its large size (**F**). (**G**) Schematic of the steps through which conventional muscles and the MOL form. The genotype of flies is *1151-GAL4/Y; UAS-mCD8::GFP*. Scale bar: 50 µm.

To better understand how the MOL forms, we employed timelapse imaging of the MOL differentiation process during the pupal stage, using GFP labeling of myoblasts with the aid of *1151-GAL4*. We found that proliferating myoblasts situated laterally migrate toward the midline and aggregate, fusing to form a myotube within 32 h after puparium formation (apf; Fig. 2; Supplementary Movie S1 online). The myotubes formed in this manner extended numerous small processes resembling filopodia until the completion of morphogenesis. Conventional muscles surrounding the MOL similarly underwent morphogenesis and terminally differentiated into adult muscles by 40 h apf (Fig. 2). In contrast, the MOL was found to grow beyond this timepoint and, at 50 h apf, it fully matured into the adult MOL (Fig. 2).

Next, we conducted timelapse imaging of the growing tip of the Mind neuron, with a particular focus on the moment of its initial contact with the MOL (Supplementary Fig. S1 online, Supplementary Movie S2 online). We found that the tip of the Mind neuron travels together with migrating myoblasts while extruding many filopodia, which actively explore (as if they search correct targets) the myotubes they encounter. Although the Mind neuron extended branches onto conventional muscles during the search period, all of these branches retracted except for the one in contact with the presumptive MOL (Supplementary Fig. S1 online). These observations imply that both the Mind neuron and MOL are actively engaged in the establishment of correct synaptic connections.

**Figure 3:**
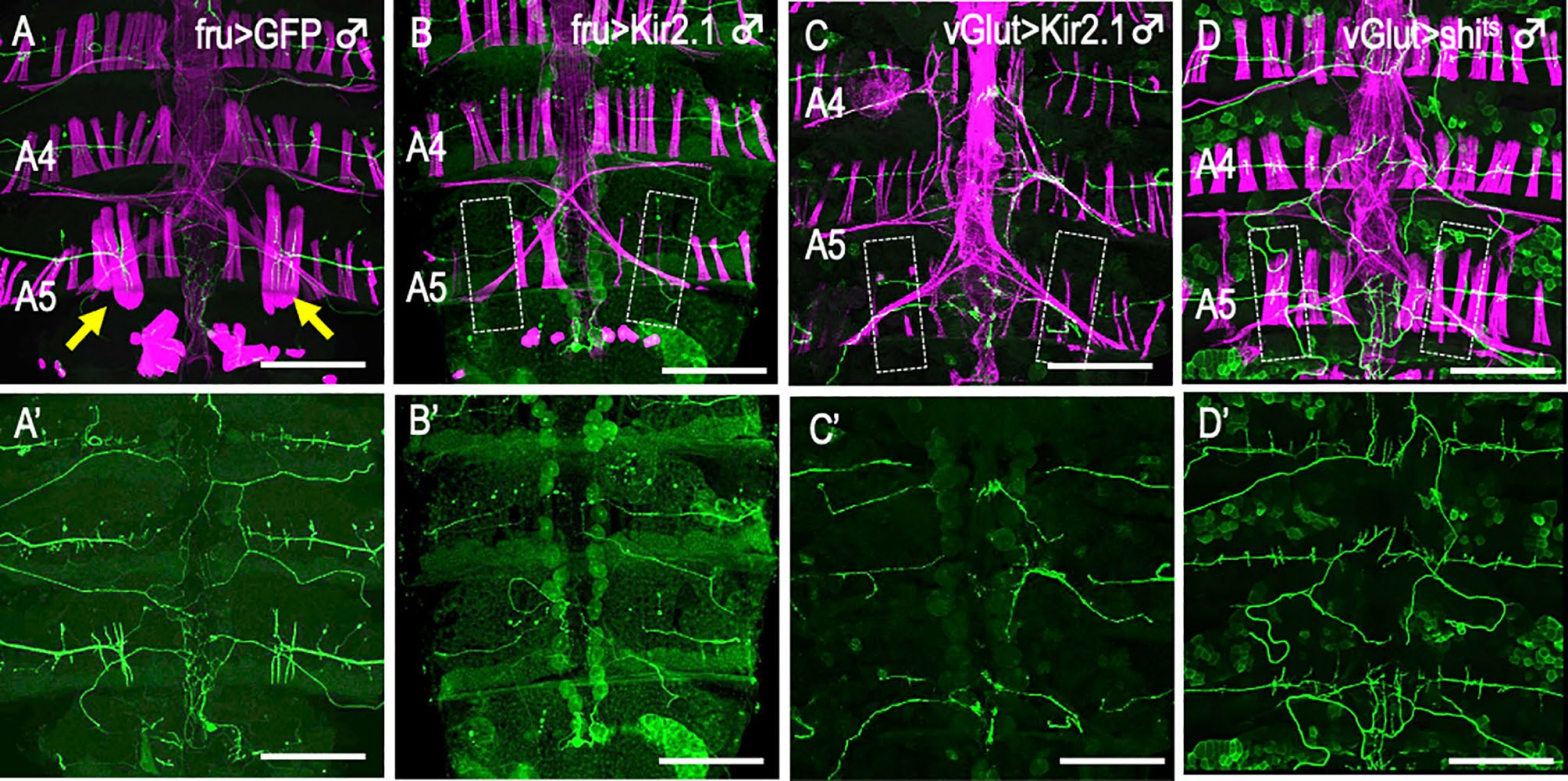
Lack of neural activity or synaptic transmission results in the loss of the MOL. (**A**–**D’**) Transgenic expression of *Kir2.1* to silence neural activity (**B**–**C’**) or *shi*^*ts*^ to block synaptic transmission (**D**,**D’**) impaired the MOL formation, while the MOLs were intact when GFP was expressed (**A**, yellow arrows). The boundaries of anticipated position of the MOL or of presumptive MOL substitutes are delineated by broken lines in (**B**–**D**). To silence neural activity with *Kir2.1*, *Tub-GAL80*^*ts*^ was inactivated after the white pupa stage by a temperature increase from 25 °C to 30 °C (**C**), and synaptic transmission was blocked with *shi*^*ts*^ by a temperature increase from 25 °C to 30 °C at 24 h apf and thereafter (**D**). The severity of the effect of *shi*^*ts*^ expression varied depending on the *shi*^*ts*^ fly line used. In this study, a *shi*^*ts*^ line with the strongest effect was used. To drive *UAS* transgenes, *fru-GAL4* (**A**,**B**) or *vGlut-GAL4* (**C**,**D**) was used. Innervating motoneurons were labeled with GFP and muscles were visualized with phalloidin (magenta). The genotypes of the flies are *y hs-flp;UAS-mCD8::GFP; fru-GAL4/TM6B* (**A**,**A’**) and *y hs-flp; UAS-mCD8::GFP/ Mef2-LexA, 13xLexAop2-6xmCherry-HA; fru-GAL4/UAS-Kir2.1* (**B**,**B’**), *y hs-flp; vGlut*^*OK371*^*-GAL4 UAS-mCD8::GFP/G13 Tub-GAL80*^*ts*^*; UAS-Kir2.1-GFP/* + (**C**,**C’**) and *y hs-flp; vGlut*^*OK371*^*-GAL4 UAS-mCD8::GFP/* + *; UAS-* × *20 shi*^*ts*^*/* + (**D**, **D’**). Scale bar: 200 µm.

To understand the mechanism by which the correct synaptic contact is selectively stabilized, we examined the effects of suppressing neural firing by the overexpression of *Kir.2.1*, an inward rectifier K^+^ channel gene under the control of *fru-GAL4* (Fig. 3B,B’; compare with A,A’). Notably, inhibition of the firing of the Mind neuron not only prevented the MOL from forming but also induced degeneration of the Mind neuron. In sharp contrast with the Mind neuron–MOL interactions, conventional muscles formed even when neural activities were suppressed by Kir.2.1 as driven by the pan-motoneuronal driver *vGlut-GAL4*, despite the fact that neural innervation was grossly aberrant under these conditions (Fig. 3C,C’; compare with Fig. 1A,A’). *vGlut-GAL4*-mediated Kir.2.1 expression in all motoneurons resulted in a loss of MOL, likely as a result of Kir.2.1 expression in the Mind motoneuron.

**Figure 4:**
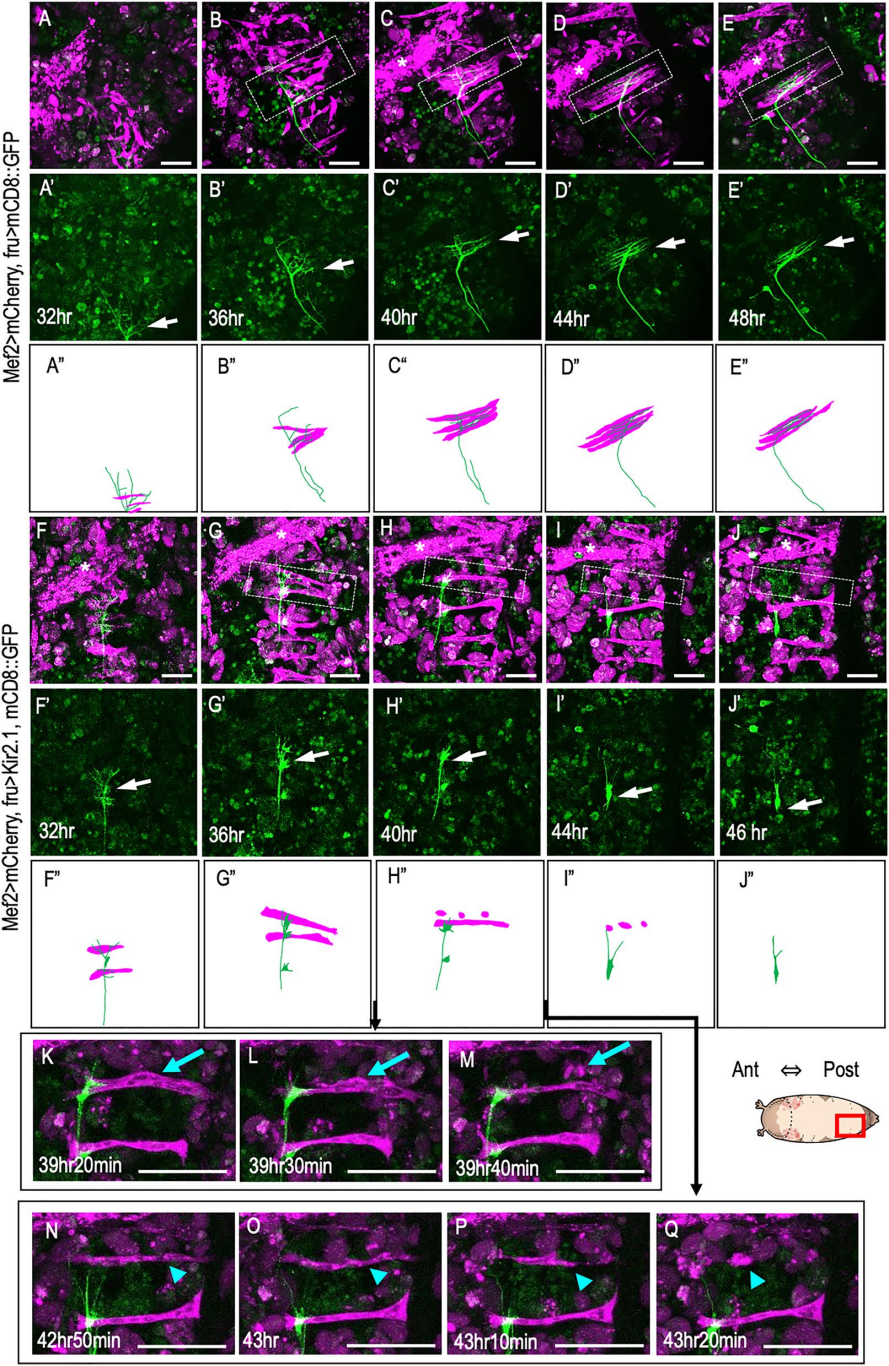
Neural activity silencing causes Mind axon retraction and MOL degeneration. (**A**–**E”**) Normal formation of the Mind neuron‒MOL synapse. The axon tip (magenta) together with myoblasts (green) migrate toward the midline (the upper side) by 36 h apf, when fine processes of the growth cone extend along the MOL (boxed with broken lines), establishing synapses on it 40–48 h apf. The growth cone of the Mind neuron is indicated by white arrows. Asterisks indicate larval muscles. Images composed of muscles and nerves (**A**–**E**), nerve only (**A’**–**E’**) and schematic drawings of the above images (**A”**–**E”**) are shown. (**F**–**J”**) Loss of the synapse upon neural silencing with Kir2.1. By 36 h apf, the Mind neuron axon extends processes on the MOL even in the absence of neural activity. Subsequently, the Mind axon tip loses fine processes at 36 h apf and is then withdrawn back to the soma at 40–46 h apf. The nerve and muscle patterns are schematically shown in (**F”**–**J”**). (**K**–**Q**) Enlarged images of the contact point of the Mind axon (green) with the MOL (magenta), captured every 10 min between 39 h 20 min and 43 h 20 min apf. Myotubes undergoing fragmentation are indicated by blue arrows and arrowheads. The genotypes of flies are *y hs-flp; Mef2-LexA, 13xLexAop2-6xmCherry-HA/ UAS-mCD8::GFP; fru-GAL4/* + for (**A**–**E’**) and *y hs-flp; UAS-mCD8-GFP/ Mef2-LexA, 13xLexAop2-6xmCherry-HA; fru-GALl4/UAS-Kir2.1-GFP* for (**F**–**Q**). Scale bar: 50 µm.

To further analyze the Mind neuron–MOL interactions during synaptogenesis (Fig. 4, Supplementary Movies S3, S4 online), we conducted observations to assess the effects of Kir.2.1 at shorter time intervals, specifically between approximately 39 and 45 h after puparium formation (apf) (Fig. 4K–Q). This timeframe coincided with the stage when, under normal conditions, the Mind neuron appeared to establish synaptic connections with the two myotubes comprising the MOL. At 39 h 40 min apf, the first myotube for the presumptive MOL innervated by the Mind neuron fragmented (Fig. 4K–M, blue arrows). Then, the second myotube fragmented at 43 h 20 min apf, along with gradual retraction of the Mind neuron axon (Fig. 4N–Q, blue arrowheads).

These results suggest that electrical activity in the Mind neuron is required for the maintenance of the MOL, while the presence of the MOL is indispensable for the persistent motor innervation by the Mind neuron axon. We conclude that mutual interactions across the presynaptic and postsynaptic cells are crucial for the maintenance of the Mind neuron‒MOL connections.

To obtain further insights into pre- and postsynaptic interactions, we examined possible effects of blocking vesicular release from the Mind neuron on MOL formation. We used pan-motoneuronal *vGlut-GAL4* to overexpress *shibire*^*ts*^ (*shi*^*ts*^), which encodes a conditional dominant-negative form of Dynamin, a protein essential for vesicle recycling. Exposure of *shi*^*ts*^-expressing pupae to a restrictive temperature (30 °C) impaired MOL formation without affecting the morphogenesis of conventional muscles (Fig. 3D,D’; compare with Fig. 1A,A’). However, the malformed MOL remained innervated by the Mind neuron even after exposure to the restrictive temperature. This result suggests that vesicular release of a signaling molecule from the presynaptic terminal of the Mind neuron is required for the normal formation of the MOL.

**Figure 5:**
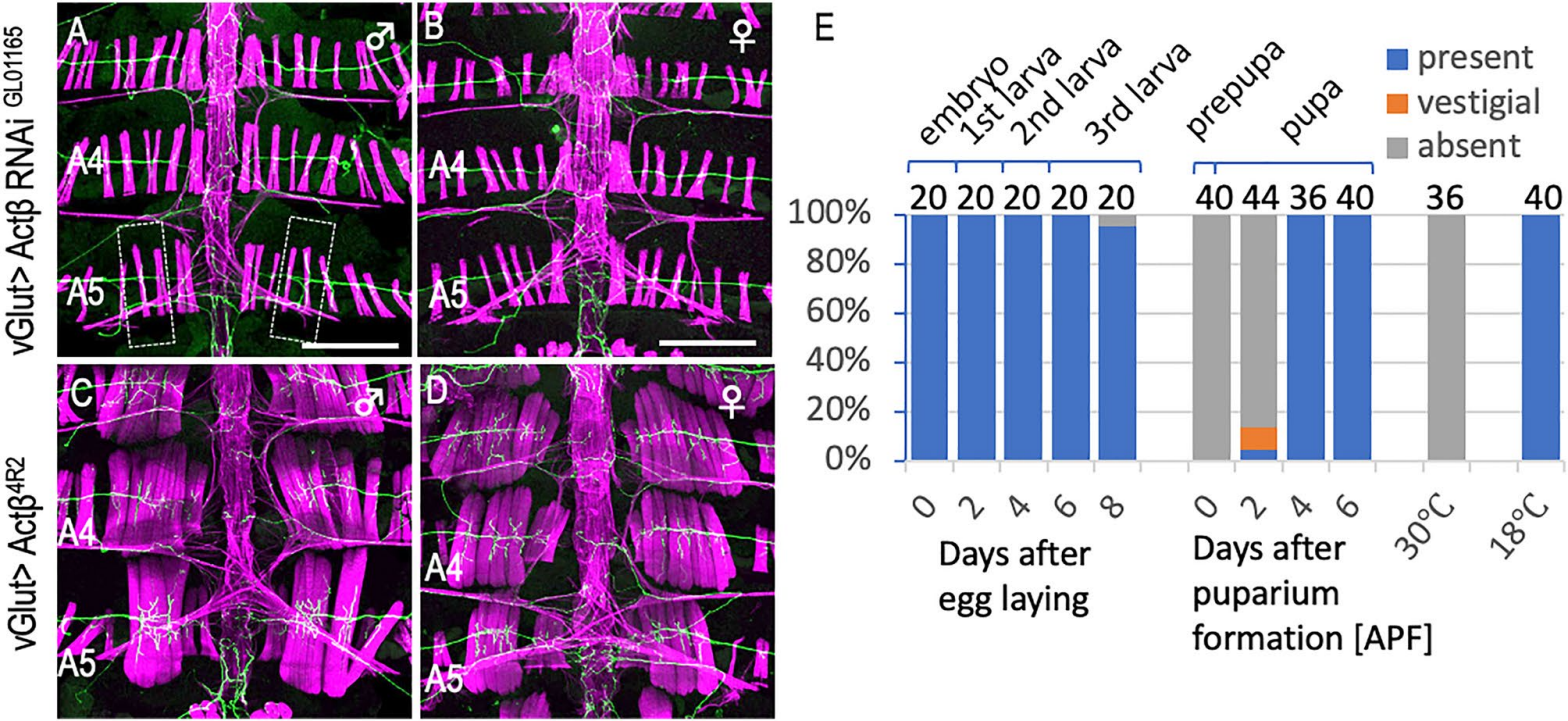
Effects of knockdown and overexpression of Actß on MOL formation. (**A**,**B**) Knockdown of *Actß* in motoneurons impaired the MOL formation in the male A5 segment (**A**) without affecting the female musculatures (**B**). (**C**,**D**) Overexpression of *Actß* induces enlargement of the MOL in male A5 and the MOL-like muscles ectopically in other abdominal segments in both sexes. (**E**) The proportion of flies exhibiting MOLs (ordinate) is shown against time (hours after egg-laying and hours after puparium formation [apf] as indicated below the bars on the abscissa). This time period involves a temperature shift from 18 to 30 °C, followed by a reversion from 30 to 18 °C. The two rightmost bars represent the values obtained from flies that experienced no temperature changes; i.e., consistently maintained at either 30 °C or 18 °C. The numerals above the bars indicate the numbers of hemisegments examined. The genotypes of files are *vGlut*^*OK371*^ > *Actß RNAi*^*GL01165*^* (y hs-flp; vGlut*^*OK371*^*-GAL4 UAS-mCD8::GFP/* + *; UAS-Actß RNAi*^*GL01165*^*/* +*)* for (**A**,**B**), *vGlut*^*OK371*^ > *Actß*^*4R2*^* (y hs-flp; vGlut*^*OK371*^*-GAL4 UASmCD8::GFP/* + *; UAS-Actß*^*4R2*^*/* +*)* for (**C**,**D**), *y hs-flp; vGlut*^*OK371*^*-GAL4/* + *, UAS-mCD8-GFP/* + *; Tub-GAL80*^*ts*^*/UAS-Actß RNAi*^*GL01165*^ for (**E**). Scale bar: 200 µm.

**Figure 6:**
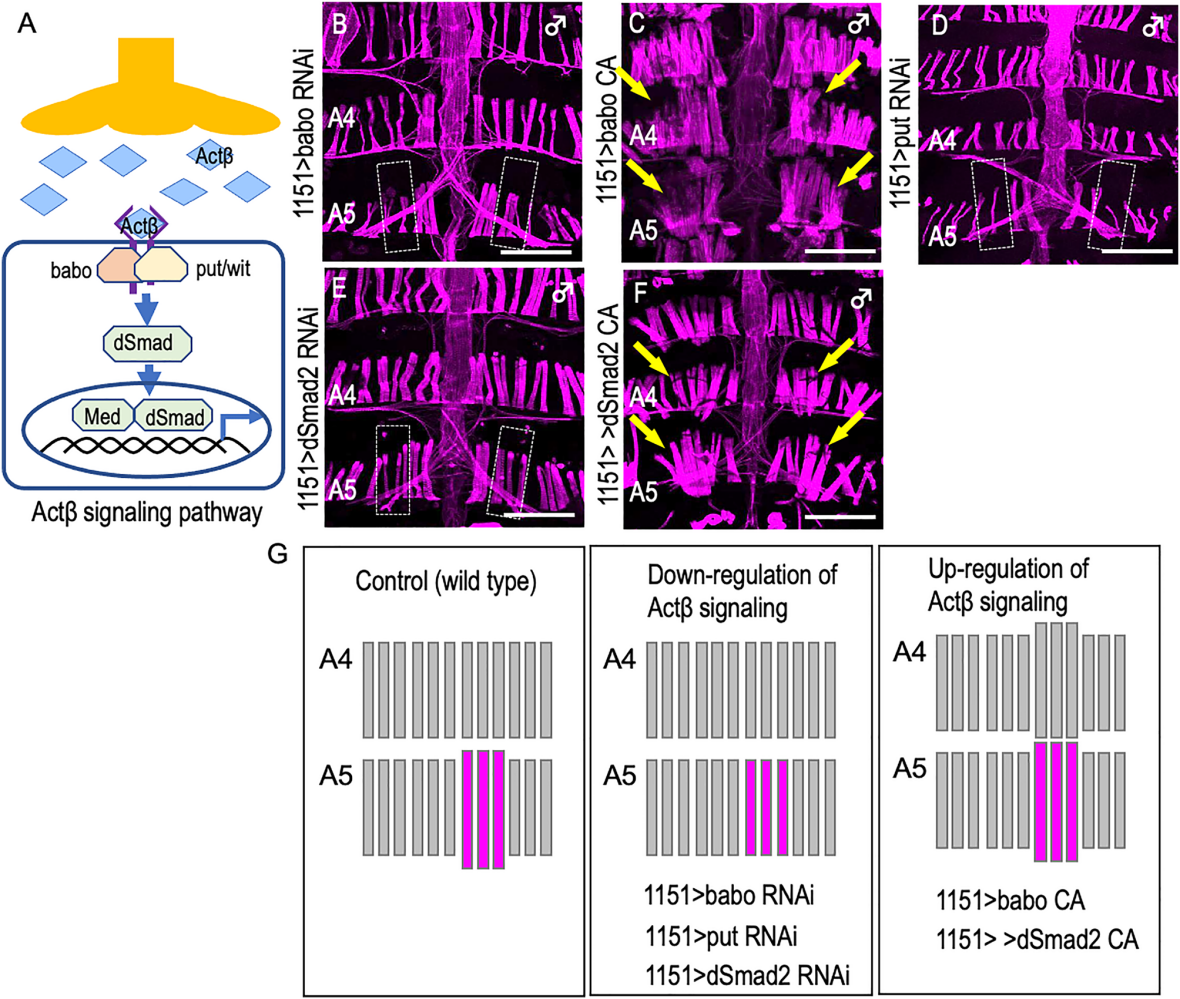
Involvement of Actß signal transduction in MOL formation. (**A**) Schematic of Actß signaling. (**B**–**F**) Knockdown of either of *babo* (**B**) or *put* (**D**), the genes for two Actß receptor components, or of the gene for dSmad2 (**E**), a downstream transcription factor results in the loss of the MOL, whereas overexpression of their constitutively active form (**C**,**F**) promotes the formation of the MOL, accompanied by ectopic induction of the MOL-like muscles in abdominal segments other than A5 in males. The MOL and MOL-like muscles are indicated by yellow arrows. Schematic drawings of the muscle structure under three different conditions are shown in (**G**). The genotypes of the flies are *1151-GAL4/Y; UAS-mCD8::GFP/UAS-babo RNAi*^*10E2/6E2*^ (**B**), *1151-GAL4/Y; UAS-mCD8::GFP/* + *; UAS-babo CA/* + (**C**), *1151-GAL4/Y; UAS-mCD8::GFP /UAS-put RNAi*^*7904R-3*^ (**D**), *1151-GAL4/Y; UAS-mCD8::GFP/* + *; UAS-sMad2 RNAi*^*JF02320*^*/* + (**E**) and *1151-GAL4/Y; UAS-mCD8::GFP/* + *; UAS-sMad2*^*SDVD (3)*^*/* + (**F**). Scale bar: 200 µm.

In an attempt to identify a signaling molecule released from the Mind neuron to induce MOL formation, we employed in vivo RNAi interference to knock down genes encoding ligands or receptors known to function in other morphogenetic events (Supplementary Table S1 online). Among the candidate ligands examined, Activin ß (Actß) was the sole molecule that affected MOL formation specifically; pan-motoneuronal overexpression of *Actß RNAi* resulted in a complete loss of the MOL (Fig. 5A,B, Supplementary Table S2 online). Overexpression of *Actß* in motoneurons with *vGlut-GAL4* caused the formation of extra MOL-like muscles in other abdominal segments in males and also in females (Fig. 5C,D, Supplementary Table S3 online). Similarly, targeted expression of RNAi against the *babo* gene encoding the type I receptor or the *put* gene encoding the type II receptor (Fig. 6A) in myoblasts completely suppressed MOL formation (Fig. 6B,D,G, Supplementary Table S4 online). Furthermore, the primary transcription factor mediating intracellular Activin actions, dSmad2 (Fig. 6A), abrogated MOL formation upon knockdown (Fig. 6E,G, Supplementary Table S4 online). Conversely, the targeted expression of *Actß*, constitutively active (CA) *babo*, or CA *dSmad2* in myoblasts induced the formation of MOL-like muscles in abdominal segments other than A5 in addition to the normal MOL in A5 of male flies (Fig. 6C,F,G, Supplementary Table S3 online). The ectopic MOL-like muscles were generated in the muscle row near the midline but not the lateral muscle row.

A pervious study provided evidence that the MOL-forming myotubes actively recruit myocytes from a proliferating cell pool, which also contributes to conventional muscles^30^. Our counts of nuclei contained in a MOL fiber of control genotypes yielded a mean ± S.E.M. of 16.8 ± 0.84, whereas a MOL fiber in flies overexpressing Actß contained 22.4 ± 0.84 nuclei (Table 1). Of note, a conventional muscle fiber carried 12.1 ± 0.39 nuclei in control flies and 15.7 ± 0.53 nuclei in flies overexpressing Actß (Table 1). We consider that elevated *Actß* signaling in the innervating neurons increases the nucleus number in a single fiber of both the MOL and conventional muscles, presumably by promoting myoblast proliferation, myocyte fusion and muscle fiber growth. The observation that the formation of ectopic MOL-like muscles is restricted to the muscle row near the midline and never occurs in the lateral row implies that neural release of Actß is not sufficient for the MOL induction, and some downstream mechanisms operating in the midline but not the lateral muscles are required for the MOL formation. We found that all conventional muscles in the flies expressing *Kir2.1*, *Actß RNAi* or *put RNAi* as driven by *vGlut-GAL4* or *1151-GAL4* appeared thinner than the counterparts in control flies. Our count of nuclei contained in single fibers of the conventional muscles revealed that the number of nuclei in *Kir2.1-*expressing flies was significantly reduced in control ones (Table 1). This observation suggests that neural activity stimulates myoblast proliferation, myocyte fusion and/or growth of muscle fibers in conventional muscles. On the other hand, we also found that the MOL but not conventional muscles was lost by inhibiting the neural activity, which implies that neural activity is required for an additional mechanism that is uniquely involved in the formation of the MOL. Apart from the specific ability of *Kir2.1* to silence neural activities, it is also possible that *Kir2.1*-expressing flies suffered from generalized defects in development, including the observed muscle undergrowth, because these manipulated flies were lethal or semi-lethal at the pharate pupal stage. In this study, a few escaper flies that remained in the pupal case were collected for examination by peeling the case at a timepoint equivalent to the post-eclosion date in other genotypes.

**Table 1:** Number of nuclei per fiber in the MOL and the conventional muscles.

	MOL	Conventional muscle
	m ± S.E.M. (n)	m ± S.E.M. (n)
*vGlut*^*OK371*^ > *mCD8::GFP* (control)	16.8 ± 0.84 (17)	12.1 ± 0.39 (19)
*vGlut*^*OK371*^ > *Actß*^*4R2*^	22.4 ± 0.84 (17)*	15.7 ± 0.53 (16)**
*vGlut*^*OK371*^ > *Kir2.1*		10.1 ± 0.38 (16)***

The number of nuclei per a fiber was counted and the mean and the standard error of the mean (m ± S.E.M.) are indicated.

The number of fibers examined is shown in parentheses.

*Significantly different from the control value for the MOL at P = 5.3 × 10^–5^ using the Student’s t-test.

**Significantly different from the control value for the conventional muscle at P = 3.6 × 10^–6^ using the Student’s t-test.

***Significantly different from the control value for the conventional muscle at P = 7.5 × 10^–4^ using the Student’s t-test.

**Figure 7:**
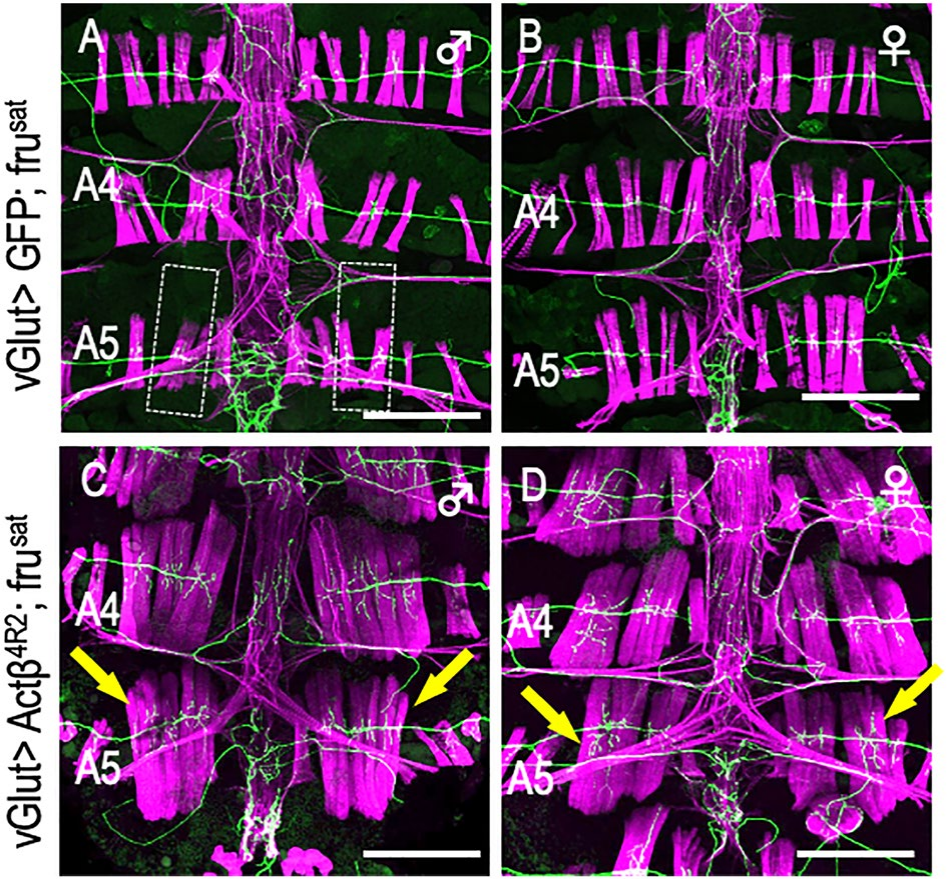
Overexpression of Actß bypasses the need of *fru* in MOL induction. Motoneuronal overexpression of Actß in a *fru*^*sat*^ homozygous male (**A**) and female (**B**) that otherwise lacks the MOL induced the MOL ((**C**) and (**D**); yellow arrows). The genotypes of flies were *y hs-flp; vGlut*^*OK371*^*-Gal4 UAS-mCD8::GFP/CyO; fru*^*sat*^*/ fru*^*sat*^ for (**A**, **B**) and *y hs-flp; vGlut*^*OK371*^*-Gal4 UAS-mCD8::GFP/ UAS-Actß*^*4R2*^*; fru*^*sat*^*/ fru*^*sat*^ for (**C**,**D**). Scale bar: 200 µm.

To pinpoint the developmental stage at which Actß is necessary for MOL formation, we restricted the Actß knockdown to a 24-h period during a distinct developmental stage. We achieved this by conditionally inactivating GAL80^ts^ at 30 °C, which is typically effective at inhibiting GAL4 at 18 °C. We found that MOL induction was abrogated only when *Actß* was knocked down in the first half of the pupal stage (~ at 48 h apf), indicating that the critical period for MOL induction by Actß resides in the early pupal stage (Fig. 5E). At this stage, both the growth cone of the Mind neuron and myocytes are migrating together from the lateral edge of the segment toward the midline, and in fact, our timelapse imaging revealed that the growth cone is in contact with myocytes during the migration. It is tempting to postulate that Actß is released by the migrating Mind neuron to predispose myoblasts to develop characteristics of the MOL at the later pupal stage. Importantly, the overexpression of *Actß* in motoneurons using *vGlut-GAL4* in the *fru* mutant background not only restored the MOL in A5, which would otherwise be MOL-less, but also led to the ectopic formation of MOL-like muscles in other abdominal segments (Fig. 7C,D; compare with A,B, Supplementary Table S5 online). We conclude that Actß derived from the Mind neuron is indispensable for the induction of the MOL and that Actß bypasses the need for FruM in the MOL formation.

## Discussion

Since its discovery, the MOL has garnered significant attention from biologists due to its unique potential for uncovering the mechanistic basis for inductive interactions among cells at a single-cell resolution. Nonetheless, the molecular identity of the signal by which the FruM-expressing Mind neuron communicates with myoblasts to form the MOL has remained unknown. In this study, we demonstrated that presynaptic Actß is an essential factor for MOL induction. Motoneuron-derived Actß has been shown to promote the growth of larval muscles in *Drosophila*^43^, contrary to the inhibitory effect of Actß on vertebrate muscles^44^. In both *Drosophila* and vertebrates, Actß signaling regulates the insulin receptor (InR)/TORC1 pathway to regulate muscle size. In this study, Actß was artificially downregulated in not only the Mind neuron but also other motoneurons, as its expression was driven by *vGlut-GAL4*. Nonetheless, only the MOL was lost; conventional muscles were spared. This may suggest the presence of additional signaling that compensates for Actß deficiencies in the neuromuscular system of conventional muscles. The *Drosophila* genome harbors a gene encoding another *Actß* family member, Myoglianin^45^. Larval muscles express Myo, a protein reported to act remotely on imaginal discs to promote their growth, likely through circulation. Myo is known to share receptors and downstream transduction pathways with Actß in imaginal discs^45^. It remains to be examined whether Myo is expressed in adult muscles and whether the loss of Myo disrupts conventional muscles in the absence of Actß.

Our study demonstrated that providing Actß through motoneurons other than the Mind neuron is enough to induce the formation of MOL-like muscles in male abdominal segments beyond A5 and in female A5, as well as in other abdominal segments. The fact that these motoneurons do not express FruM indicates that Actß overexpression can bypass the FruM-dependent mechanism necessary for MOL induction. This raises the possibility that FruM is required in the Mind neuron to boost *Actß* expression. Indeed, *Actß* has been suggested to be a transcriptional target of FruBM, as indicated by findings from a whole brain sample^46^. Single-cell profiling of gene transcription for the Mind neuron will be a straightforward approach toward the identification of FruM in this cell.

Our experiments to suppress motoneuronal electrical activities demonstrated that the Mind neuron retracts its axon in the absence of neural activity, and other motoneurons fail to maintain their innervation on conventional muscles. This could mean that it was neural silencing per se that induced axonal degeneration of the Mind neuron. Alternatively, the loss of the MOL might deprive one or more MOL-derived neurotropic factors that otherwise protect the Mind neuron axon from degeneration. These two mechanisms may even operate in synergy. In fact, neural activity in presynaptic neurons has been shown to stimulate the retrograde trafficking of neurotrophic factors derived from postsynaptic cells in both hippocampal and *Drosophila* motoneurons^47^. One may envisage that retrograde transport of muscle-derived Actß toward the neuronal soma will be impaired if neural activity is blocked by Kir2.1, leading to a failure in transcription de novo for the maintenance of the Mind neuron^48^. This hypothesis awaits an experimental test.

Hence, the present study points to the possibility that FruM exerts cell non-autonomous actions on neighboring cells through trophic interactions via its transcriptional targets. The MOL itself does not express FruM, yet its formation relies on the presence of FruM in the innervating motoneuron (thus this FruM action is cell non-autonomous for the MOL), whose survival in turn depends on the presence of the MOL. This cell non-autonomous action of FruM occurs in addition to its cell-autonomous role in determining the identity of the cell that expresses FruM. These trophic interactions can be bidirectional, meaning that the state of the FruM-expressing cell can, in turn, be influenced by the state of its interacting partner cell. This non-autonomous action of FruM within cells introduces a higher layer of complexity to neuronal wiring, offering a flexible mechanism for reorganizing circuitry to adapt to changes in behavioral outputs. Uncovering the regulation of this cellular plasticity at the molecular and genomic levels is an urgent matter.

## Methods

### Fly strains

Flies were reared on cornmeal-yeast medium at 25 °C under constant illumination, unless mentioned otherwise. A *fru-Gal4* line was a gift from B.J. Dickson. A *fruP1-LexA* line was provided by B. Baker. A line of *1151-Gal4* was generously provided by K. VijayRaghavan. The following strains were obtained from the Bloomington Stock Center: 1*3xLexAop2-6XmCherry-HA}VK00018/CyO; Dr[1] /TM6C, Sb[1]Tb[1]* (BDSC 52272), *w; Mef2-Gal4/CyO *(BDSC 54872)*, 20X UAS -shi*^*ts1*^ (BDSC 66600)*, w; UAS-kir2.1-GFP* (BDSC 6595), *w*^*1118*^*; vGlut*^*OK371*^ (BDSC 26160), w[*]; P{w[+ *mC]* = *tubP-GAL80[ts]}20; TM2/TM6B, Tb[1]* (BDSC 7019). To identify possible factors that induce the MOL, we screened components of several known signaling pathways, employing in vivo RNA interference techniques. To examine the effects of ligand or receptor knockdown on MOL formation, female flies of the genotypes *y hs-flp; vGlut*^*OK371*^*-Gal4, UAS-mCD8::GFP* for neural expression or *1151-Gal4; UAS-mCD8::GFP* for muscular expression were crossed with male flies of *UAS-ligand RNAi* or *UAS-receptor RNAi* counterparts, respectively. After eclosion, their progeny were dissected to examine whether the MOL had formed. In cases of pharate lethal flies, their pupal opercula were excised during the pharate stages to facilitate eclosion, or pharate flies were dissected manually. The stocks employed in our screening are inventoried in Supplementary Table S1 online. Other fly strains related to the Activin signaling pathway include *UAS-Smad2 RNAi *^*JF02320*^ (BDSC 26756), *UAS-Smad2 RNAi *^*GL01476*^ (BDSC 43138), and *UAS-Smad2 RNAi *^*HMJ30064*^ (NIG HMJ30064). Additional lines, i.e., *UAS-dSmad2*^*SDVD (2)*^*, UAS-dSmad2*^*SDVD (3)*^*, and UAS-Actß*^*4R2*^ were generously provided by M. O’Connor.

### Histology

For the observation of the abdominal muscles and their motoneuron terminals, the dissected abdomens were fixed with 4% formaldehyde solution for 30 min. Immunohistochemical staining was then performed, utilizing a primary anti-GFP antibody (rabbit polyclonal, 1:500; Molecular Probes A6455) followed by a secondary application of Alexa Fluor 488 goat anti-rabbit IgG (1:500; Invitrogen A11034). Acti-stain™ 555 phalloidin (CYTOSKELETON, PHDH1-A) or Acti-stain™ 448 (CYTOSKELETON, PHDG1-A) was employed to stain the muscles. DAPI (1:500; Dojindo) was used to stain the muscle nuclei. Images were obtained using a fluorescent microscope (Olympus AX70), a Zeiss confocal microscope (LSM980 with Airyscan2) with ZEN software, or a Leica confocal microscope (TCS SPE) with LAS. X software. Each abdominal hemisegment was examined to determine the presence or absence of MOLs. MOLs are distinguishable from conventional muscles based on the anterior/posterior displacement of its attachment sites on the tergite. If muscles in the location of the MOL exhibited clustering or excessive growth either anteriorly or posteriorly, the MOL was classified as vestigial.

### Temperature control for experiments with temperature-sensitive flies

Female flies of the *vGlut*^*OK371*^*-Gal4, UAS-mCD8-GFP; Tub-Gal80*^*ts*^ genotype were crossed with *UAS-Actß RNAi* male flies and allowed to lay eggs for a period of 3 h at 18 °C. Larvae were raised at 18 °C until specified developmental time points (0, 2, 4, 6, and 8 days after egg-laying) and then transferred to a temperature of 30 °C for 24 h, followed by a return to the ambient temperature of 18 °C until they reached adulthood. The offspring, including white pupae at 0 days, and those at 2, 4, and 6 days after puparium formation (apf) at 18 °C, were carefully placed within a moist chamber. The chamber was then transferred to an incubator at 30 °C for 24 h, after which the temperature was decreased to 18 °C until eclosion. During the pharate pupal stages, the pupal operculum was meticulously removed. The emerged adult flies, aged for more than 24 h, were dissected to expose their abdominal musculature, which was fixed with 3.7% formaldehyde solution and subsequently stained with Acti-stain™ 555 phalloidin (CYTOSKELETON, PHDH1-A). Each abdominal hemisegment was examined under an Olympus AX70 fluorescence microscope to ascertain the presence or absence of MOLs. The normalization of the heating rate was adjusted to account for the developmental stage duration at 25 °C, effectively doubling the duration of development at 18 °C relative to that at 25 °C.

Because *Kir2.1* expression as driven by *vGlut-GAL4* was lethal to individuals at stages earlier than the white pupa stage, *GAL80*^*ts*^ was used to restrict *Kir2.1* transcription to later pupal stages.

### Live imaging

Live imaging of developing abdominal muscles and/or neurons during the pupal stage was conducted from 24 h apf to the desired developmental stages, as detailed by Nojima et al.^42^. The pupal puparium was carefully removed at 24 h apf and placed onto a coverslip to facilitate adhesion. Subsequently, the cover glass, with the pupa mounted in a side-up orientation, was affixed to a tissue culture slide (Lab-Tek). Time-lapse imaging of labeled fluorescein markers was performed at 10-min intervals using a Leica TCS SPE confocal microscope.

### Statistical analysis

All statistics were performed using Excel ver.16.54. Statistical significance was evaluated by the Student t-test (tow-tailed). A p value of less than 0.01 was considered to be statistically significant.

### Supplementary Information

Supplementary Video 1.

Supplementary Video 2.

Supplementary Video 3.

Supplementary Video 4.

Supplementary Information.

## Data Availability

The datasets used for each figure are provided in the source data of this paper. Further information can be requested from the corresponding authors.
