# Supplementary material for: Activin is a neural inducer of a male-specific muscle in Drosophila
Source: Sci Rep. 2024 Feb 14;14:3740. doi: 10.1038/s41598-024-54295-3 (PMC10866940; doi:10.1038/s41598-024-54295-3)
Supplement: Supplementary file 5 — Supplementary Information. [file 41598_2024_54295_MOESM5_ESM.pdf]

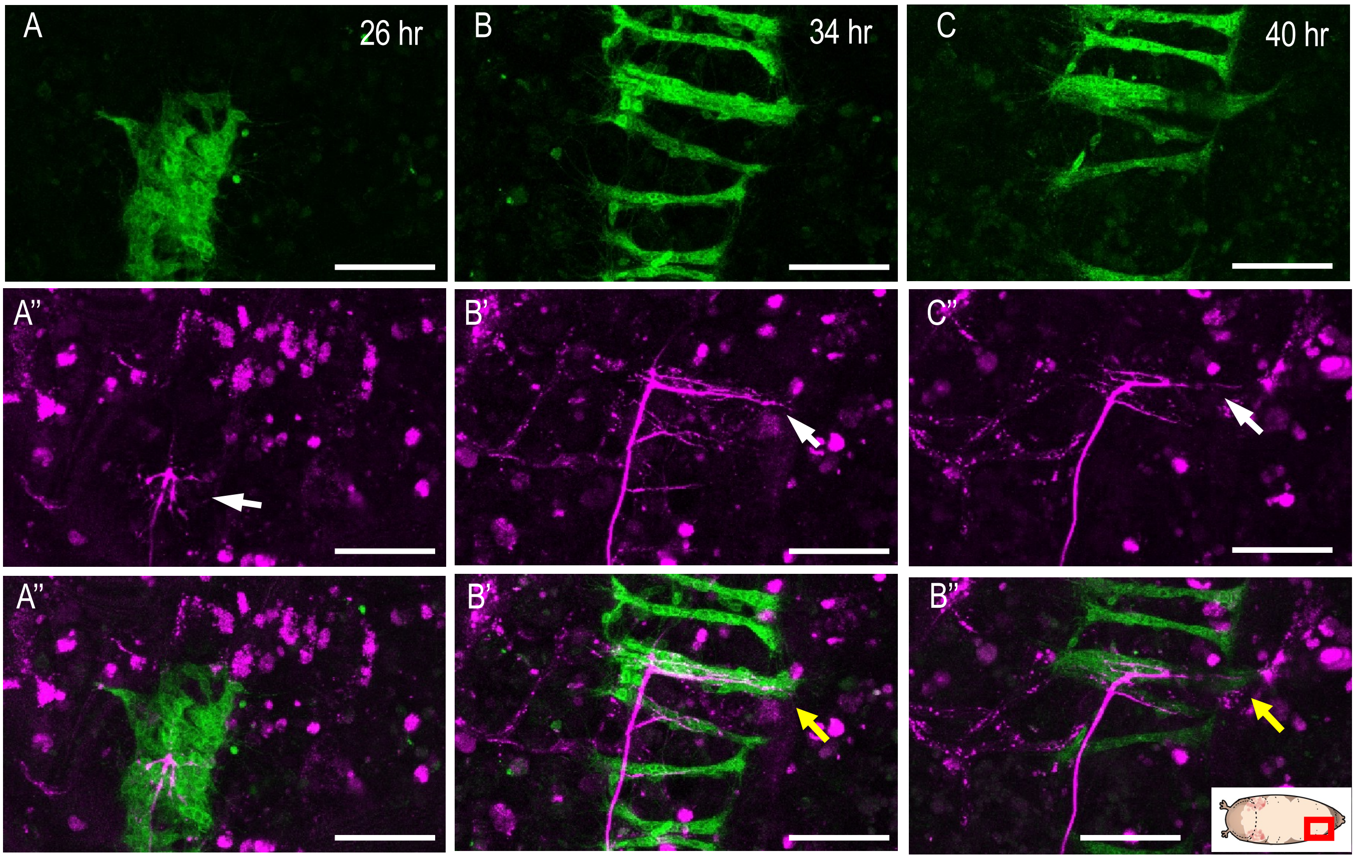

Supplementary Fig. S1 The Mind neuron axon terminal in the process of contacting the MOL.

(**A-C"**) Timelapse images of muscles (**A-C**, **A"-C"**; green) and the Mind neuron (**A'-C'**, **A"-C"**, magenta) at 26 (**A**, **A'**, A"), 34 (**B**, **B'**, **B"**), and 40 (**C**, **C'**, **C"**) hr apf. The Mind neuron and MOL are indicated by white and yellow arrows, respectively. The Mind neuron axon tip that actively extends filopodia migrates together with a group of myoblasts from the lateral side (the lower side) to the midline (the upper side). The Mind axon then extends processes along the myotubes that subsequently develop into the MOL and forms synapses on them. The genotypes of the flies are *1151-GAL4/Y; UAS-mCD8::GFP/13xLexAop2-6xmCherry-HA; fruP1-LexA/+*. Scale bar: 50 µm.

Supplementary Table S1 The list of MOL-inducer candidates tested in screens with RNAi knockdown

signaling pathway									
ligand[1]	stock no.[2]	name	MOL[3]	development[4]	receptor[5]	stock no.[2]	name	MOL[3]	development[4]
**Activin signaling pathway**									
Actß	BL 29597	*Actß RNAi* HMS00799	loss	+	bab	BL 25933	*babo RNAi* JF01953	loss	+
	BL 42493	*Actß RNAi* HMJ02057	loss	+		BL 44401	*babo.b RNAi*	loss	ph.l.*
	BL 42795	*Actß RNAi* GL01165	loss	semi ph.l.		O'Connor	*babo.b RNAi* 10E2/6E2	loss	ph.l.*
	NIG 11062R-1	*Actß RNAi* 11062R-1	loss	+		O'Connor	*babo RNAi* 1A3/58A5	loss	ph.l.*
	NIG 11062R-2	*Actß RNAi* 11062R-2	loss	+		NIG HMS02033	*babo RNAi* HMS02033	+	+
						BL 41585	*babo RNAi* GL00702	+	+
daw	NIG HMS01110	*daw RNAi* HMS01110	+	+		BL 44400	*babo.c RNAi*	+	+
						BL 44402	*babo.a RNAi*	+	+
myo	NIG 1838R-1	*myo RNAi* 1838R-1	+	+					
					put	NIG 7904R-2	*put RNAi* 7904R-2	+	+
						NIG 7904R-3	*put RNAi* 7904R-3	loss	ph.l.*
						NIG GL00069	*put RNAi* GL00069	+	+
						BL 27514	*put RNAi* JF02664	loss	ph.l.*
						BL 39025	*put RNAi* HMS01944	+	+
					wit	NIG 10776R-1	*wit RNAi* 10776R-1	+	+
						NIG 10776R-2	*wit RNAi* 10776R-2	+	+
						BL 25949	*wit RNAi* JF01969	+	+
						BL 41906	*wit RNAi* HMS02298	+	+
**Notch signaling pathway**									
Dl	BL 33655	*Dl RNAi* HMS00061	loss	ph.l.	N	NIG 3936R-3	*N RNAi* 3936R-3	small	adult weak
	BL 28616	*Dl RNAi* JF03031	+	+		NIG GL 00092	*N RNAi* GL00092	small	semi-ph.l.*
	NIG HMS01309	*Dl RNAi* HMS 01309	small	ph.l.					
Ser	NIG HMS01179	*Ser RNAi* HMS01179	+	+					
	BL 28616	*Ser RNAi* JF03031	+	+					
	BL 33655	*Ser RNAi* HMS00061	loss	semi-ph.l.					

**FGFR signaling pathway**									
bnl	NIG 4608R-1	*bnl RNAi*$^{4608R-1}$	+	+	btl	NIG 32134R-1	*btl RNAi*$^{32134R-1}$	+	+
						NIG 32134R-2	*btl RNAi*$^{32134R-2}$	+	+
pyr	NIG HMJ30113	*pyr RNAi*^{HMJ30113}	+	+		NIG HMS02038	*btl RNAi*^{HMS02038}	+	+
ths	NIG HMJ22427	*ths RNAi*^{HMJ22427}	+	+	htl	NIG HMJ22375	*htl RNAi*^{HMJ22375}	abnormal	ph.l.
						NIG HMS01437	*htl RNAi*^{HMS01437}	+	+
**Hedgehog signaling pathway**									
hh	NIG 4637R-2	*hh RNAi*$^{4637R-2}$	+	+	smo	NIG 11561R-1	*smo RNAi*$^{11561R-1}$	+	+
	NIG HMS00492	*hh RNAi*^{HMS00492}	+	+		NIG HMJ30064	*smo RNAi*^{HMJ30064}	abnormal	ph.l.
					ptc	NIG 2411R-1	*ptc RNAi*$^{2411R-1}$	+	+
						NIG HMJ22288	*ptc RNAi*^{HMJ22288}	+	+
**Wnt-TCF signaling pathway**									
wg	NIG HMS00794	*wg RNAi*^{HMS00794}	+	+					
	NIG 4889R-3	*wg RNAi*$^{4889R-3}$	+	+					
	NIG 4889R-4	*wg RNAi*$^{4889R-4}$	+	+					
**BMP signaling pathway**									
dpp	NIG 9885R-2	*dpp RNAi*$^{9885R-2}$	+	+	tkv	NIG 14026R-1	*tkv RNAi*$^{14026R-1}$	+	+
	NIG JF02455	*dpp RNAi*^{JF02455}	+	+		NIG 14026R-3	*tkv RNAi*$^{14026R-3}$	+	+
						NIG HMS02185	*tkv RNAi*^{HMS02185}	+	+
**EGFR signaling pathway**									
					Egfr	NIG 10079R-1	*Egfr RNAi*$^{10079R-1}$	+	adult weak
						NIG 10079R-2	*Egfr RNAi*$^{10079R-2}$	+	+

[1]ligand: Flies carrying *UAS-ligand RNAi* were crossed with flies of the genotype *y hs-flp; vGlut*^{OK371}*-GAL4, UAS-mCD8-GFP*, and flies of the genotype *vGlut*^{OK371}*>ligand RNAi* were chosen in the F1 progeny and subjected to phenotypic examinations.

[2]stock no., BL: Bloomington Drosophila Stock Center, NIG: National Institute of Genetics

[3]MOL, +: normal MOL was found in A5. abnormal: many conventional muscles were malformed.

[4]development, ph.l.: pharate lethal, ph.l.*: Conditional pharate lethal: flies emerged when their opercula were removed prior to eclosion.

[5]receptor: Flies carrying *UAS-receptor RNAi* were crossed with flies of the genotype *1151-GAL4; UAS-mCD8-GFP*, and flies exhibiting the genotype *1151> receptor RNAi* were chosen in the F1 progeny and subjected to phenotypic examinations.

SupplementaryTable S2 Effects of RNAi-mediated knockdown of the *Actß* gene on MOL formation

	A5 MOL							
	male				female			
$vGlut^{OK371}$ >	+	±	−	total*	+	±	−	total*
Actß RNAi ^{GL01165}	0	0	18	18	0	0	12	12
Actß RNAi ^{MHJ02057}	0	6	6	12	0	0	8	8
Actß RNAi $^{11062R\text{-}1}$	0	0	10	10	0	0	6	6
Actß RNAi $^{11062R\text{-}2}$	0	0	10	10	0	0	6	6

* No of hemisegments examined

SupplementaryTable S3 Effects of ectopic expression of *Actß*-related genes on muscle formation

	Extra MOL-like muscles							
	male				female			
$vGlut^{OK371}$ >	+	±	−	total*	+	±	−	total*
$Actß^{4R2}$	8	0	0	8	8	0	0	8
1151 >								
babo-CA ^{Q302D}	10	0	0	10	6	0	0	6
dSmad2 $^{SDVD\ (2)}$	0	6	2	8	0	4	2	6
dSmad2 $^{SDVD\ (3)}$	2	7	1	10	0	6	0	6

* No of hemisegments examined

SupplementaryTable S4 Effects of RNAi-mediated knockdown of *Actß*-related genes on MOL formation

	A5 MOL							
	male				female			
1151 >	+	±	−	total*	+	±	−	total*
put RNAi $^{7904R\text{-}3}$	0	0	12	12	0	0	6	6
put RNAi ^{JF02664}	0	0	32	32	0	0	6	6
babo RNAi $^{10E2/6E2}$	0	0	10	10	0	0	6	6
babo RNAi $^{1A3/58A5}$	0	0	8	8	0	0	6	6
Smox RNAi ^{GL01476}	0	0	10	10	0	0	6	6
Smox RNAi ^{JF02320}	0	0	18	18	0	0	6	6

* No of hemisegments examined

SupplementaryTable S5 Effects of ectopic expression of the *Actß* gene on muscle formation in fru^{sat} mutants

	A5 MOL & Extra MOL-like muscles							
	male				female			
$vGlut^{OK371}$ >	+	±	−	total*	+	±	−	total*
mCD8::GFP; fru^{sat}/fru^{sat}	0	0	10	10	n.d.**			
$Actß^{4R2}$*;* fru^{sat} */TM6b*	12	0	0	12	8	0	0	8
$Actß^{4R2}$*;* fru^{sat}/fru^{sat}	12	0	0	12	12	0	0	12

* No of hemisegments examined ** not determined

Supplementary Movie S1. Dynamics of the developing MOL.

Timelapse movie of myogenesis in A5 from 24 to 44 hr apf. The genotype of flies is *1151-GAL4/Y; UAS-mCD8::GFP*.

Supplementary Movie S2. The Mind neuron axon terminal in the process of contacting the MOL.

Timelapse movie of muscles (green) and the Mind neuron (magenta) from 24 to 44 hr apf. The genotype of the flies is *1151-GAL4/Y; UAS-mCD8::GFP/13xLexAop2-6xmCherry-HA; fruP1-LexA/+*.

Supplementary Movie S3. Steps toward Mind axon synaptogenesis and MOL formation.

Timelapse movie of muscles (magenta) and the Mind neuron (green) from 32 to 48 hr apf. The genotype of flies is *y hs-flp; Mef2-LexA, 13xLexAop2-6xmCherry-HA/ UAS-mCD8::GFP; fru-GAL4/+*.

Supplementary Movie S4. Steps toward Mind axon retraction and MOL degeneration following neural activity silencing.

Timelapse movie of muscles (magenta) and the Mind neuron (green) from 32 to 48 hr apf. The genotype of flies is *y hs-flp; UAS-mCD8-GFP/ Mef2-LexA, 13xLexAop2-6xmCherry-HA; fru-GALl4/UAS-Kir2.1-GFP.*
